# mTOR/autophagy pathway in the hippocampus of rats suffering intermittent hypoxia preconditioning and global cerebral ischemia-reperfusion

**DOI:** 10.18632/oncotarget.15058

**Published:** 2017-02-03

**Authors:** Ya-Ning Zhao, Xiang-Fei Guo, Jian-Min Li, Chang-Xiang Chen, Shu-Xing Li, Cheng-Jing Xu

**Affiliations:** ^1^ Nursing and Rehabilitation College, North China University of Science and Technology, 063000, China; ^2^ The Neurosurgery of Affiliated Hospital, North China University of Science and Technology, 063000, China

**Keywords:** OSAHS-patterned hypoxia, cerebral ischemia-reperfusion, mTOR, autophagy

## Abstract

We explored the role of mTOR/autophagy pathway in the aggravation of cerebral ischemia-reperfusion nerve injury caused by intermittent hypoxia. Eighty male wistar rats were divided into four groups by the random number method: sham operation group (SO group, n=20), cerebral ischemia-reperfusion group (I/R group, n=20), intermittent hypoxia and cerebral ischemia-reperfusion group (IH+I/R group, n=20), intermittent hypoxia and cerebral ischemia-reperfusion group plus mTOR inhibitor group (inhibitor group, n=20). The results showed that compared with the SO group, HE staining showed structural damage of neurons at each time point, the immunohistochemical assay showed an increasing number of mTOR and beclin1 immune-positive cells (P<0.05) and RT-PCR showed enhanced expression of mTOR and beclin1 protein in the I/R group (P<0.05). Compared with the I/R group, HE staining showed exacerbating structural damage of neurons at each time point, the immunohistochemical assay showed an increasing number of mTOR and beclin1 immune-positive cells (P<0.05) and RT-PCR showed enhanced expression of mTOR and beclin1 protein in the IH+I/R group (P<0.05). Compared with the IH+I/R group, HE staining showed remissive structural damage of neurons at each time point, the immunohistochemical assay showed a decreasing number of mTOR immune-positive cells and a rising number of beclin1immune-positive cells (P<0.05) and RT-PCR showed weakened expression of mTOR protein and enhanced expression of beclin1 protein in the inhibitor group (P<0.05). Thence, the present study indicated that intermittent hypoxia preconditioning can aggravate the nerve injury of the global cerebral ischemia-reperfusion model, and the mechanism is associated with the activation of mTOR/autophagy pathway.

## INTRODUCTION

Obstructive sleep apnea-hypopnea syndrome (OSAHS) is an independent risk factor of cerebral arterial thrombosis, which can aggravate the cerebral injury caused by cerebral arterial thrombosis [1–2]. Autophagy, which widely exists in cerebrovascular diseases such as cerebral arterial thrombosis, exerts significant effects on the pathological process of cerebral arterial thrombosis via regulating apoptosis of nerve cells [3–4]. Mammalian target of rapamycin (mTOR) is an evolutionarily conserved serine/threonine protein kinase, which can be stimulated by various factors such as growth factors, nutriments, etc., and then phosphorylate downstream target proteins to engage in genetic transcription and protein expression and affect autophagy [5–6]. Studies have shown that rapamycin can lessen hypoxic-ischemic brain damage of new-born rats, in which mTOR plays an important part [7]. ROS, oxidative stress, and inflammation responses, etc., aggravate OSAHS complicated with cerebral arterial thrombosis, which all can result in autophagy of nerve cells in a direct or indirect manner. Therefore, we speculate that mTOR/autophagy-associated pathways play an important role in the pathological process of the aggravation of cerebral ischemia nerve injury caused by intermittent hypoxia. In this study, we used rats with intermittent hypoxia preconditioning to prepare global cerebral ischemia-reperfusion models, observed the expression of mTOR/autophagy pathway and the loss and changes of nerve cells in the rat hippocampus and investigated its role in the aggravation of cerebral ischemia-reperfusion neuron injury caused by intermittent hypoxia, so as to provide experimental basis for the prevention and cure of OSAHS complicated with ischemic cerebrovascular diseases.

## RESULTS

### HE staining

**Figure 1:**
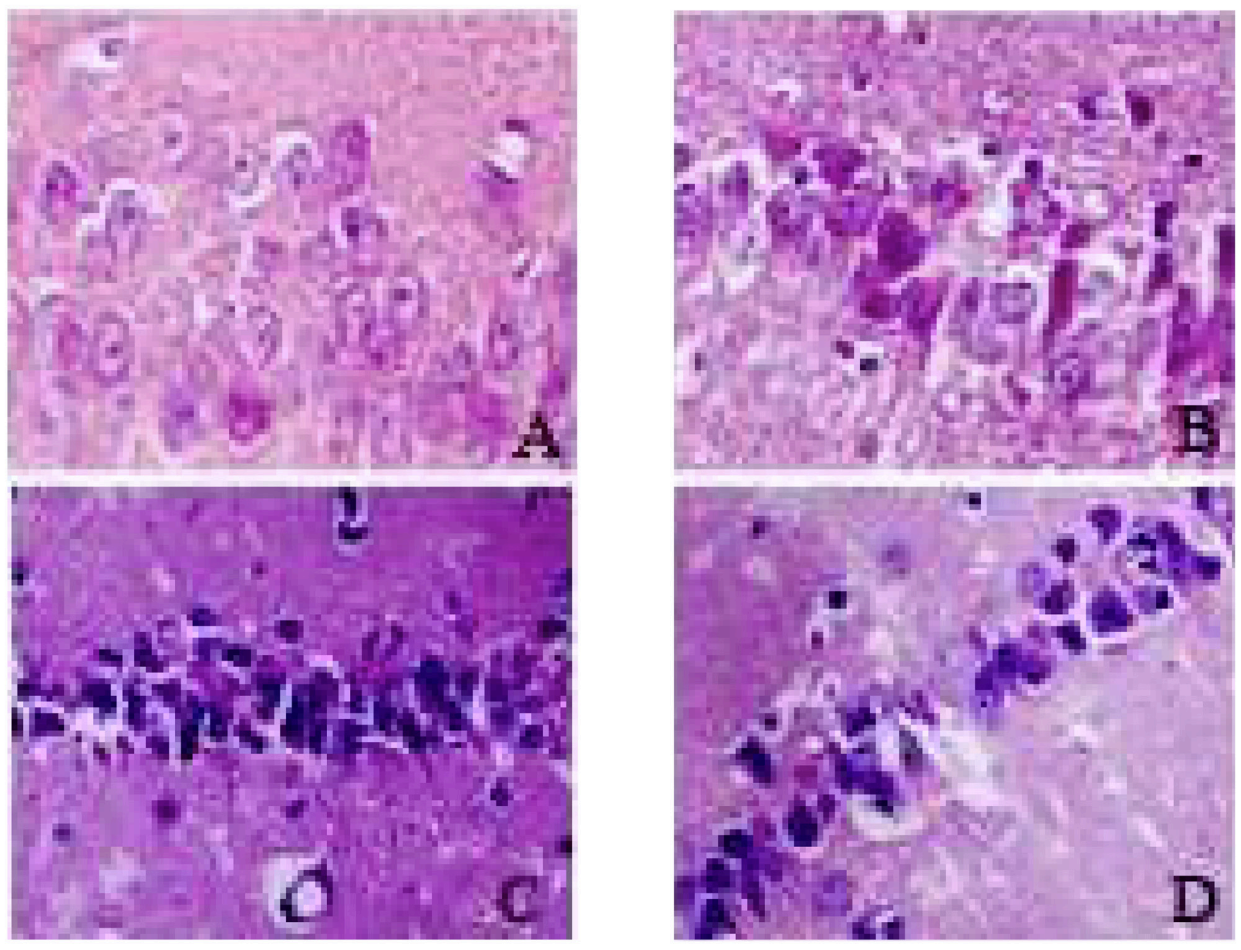
Hippocampus neurons of each group 24h after cerebral ischemia (HE staining, 400x) **A**. SO group; **B**. I/R group; **C**. IH+I/R group; **D**. inhibitor group.

The hippocampus nerve cells of the SO group showed orderly arrangement, normal structure, clear nuclei and distinct nucleoli. The I/R group showed many swollen neurons with loosen structure and deep-dyed and pyknotic nuclei, with a part of neurons completely losing their nuclei, forming a vacuolus-like structure, and showed a markedly decreased density of survival neurons at each time point (6h and 24h) (P<0.05). The IH+I/R group showed even more seriously damaged structure of neurons, more denatured and necrotic neurons with loosen structure, deep-dyed and pyknotic nuclei in neurons with a part of neurons completely losing their nuclei, forming a vacuolus-like structure, and showed a further diminished density of survival neurons at each time point (6h and 24h) (P<0.05). Compared with the IH+I/R group, the inhibitor group showed a lower degree of structural damage of neurons as well as an increased survival density of neurons at each time point (6h and 24h) (P<0.05). (See Table 1 and Figure 1)

**Table 1:** Intergroup comparison of the survival rates of hippocampus neurons (n=5, %)

Groups	n	Survival rate of neuron	
		6h	24h
SO group	5	99.08±0.84	98.65±0.75
I/R group	5	78.65±1.43*	71.71±1.39*
IH+I/R group	5	49.06±1.70*^△^	45.12±1.51*△
Inhibitor group	5	55.03±1.63*^△Δ^	50.32±1.42*^△Δ^

Note: *P<0.05, compared with the SO group; ^△^*P*<0.05, compared with the I/R group; ^Δ^*P*<0.05, compared with the IH+I/R group

### Immunohistochemical staining

#### mTOR

**Figure 2:**
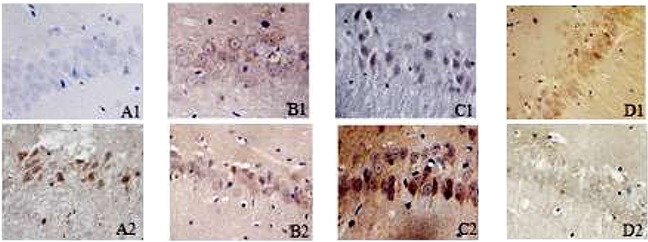
Positive expression of mTOR or beclin1 in the hippocampus of each group 24h after cerebral ischemia (immunohistochemical staining, 400x) **A1–D1**: immunohistochemical results of beclin1 in the hippocampus for SO, I/R, IH+I/R and inhibitor group respectively. **A2–D2**: immunohistochemical results of mTOR in the hippocampus for SO, I/R, IH+I/R and inhibitor group respectively.

mTOR manifests as a brown color after immuno-histochemical staining, and it is located in cytoplasm and primarily expressed in neurons. mTOR immune-positive cells were rarely seen in the SO group; compared with the SO group, the I/R group showed an increased number of mTOR immune-positive cells at each time point (P<0.05); compared with the I/R group, the IH+I/R group also showed an increased number of mTOR immune-positive cells at each time point (P<0.05); compared with the IH+I/R group, the inhibitor group showed a decreased number of mTOR immune-positive cells at each time point (P<0.05). (See Table 2 and Figure 2)

**Table 2:** Intergroup comparison of the counts of mTOR or beclin1 immune-positive cells in the hippocampus (*±s*, cells/visual field at high magnification)

Groups	n	mTOR		beclin1	
		6h	24h	6h	24h
SO group	5	14.65±0.48	15.40±0.58	2.06±0.23	2.10±0.30
I/R group	5	22.38±0.46*	24.16±0.60*	8.58±0.58*	10.58±0.49*
IH+I/R group	5	30.40±0.43*^△^	32.86±0.50*^△^	15.57±0.57*^△^	18.78±0.43*△
Inhibitor group	5	26.60±0.37*^△Δ^	28.51±0.52*^△Δ^	21.74±0.51*^△Δ^	24.32±0.49*^△Δ^

Note: *P<0.05, compared with the SO group; ^△^*P*<0.05, compared with the I/R group; ^Δ^*P*<0.05, compared with the IH+I/R group

#### Beclin1

Beclin1 manifests as a brown color after immuno-histochemical staining, and it is located in cytoplasm and primarily expresses in neurons. Beclin1 immune-positive cells were rarely seen in the SO group; compared with the SO group, the I/R group showed an increased number of beclin1 immune-positive cells at each time point (P<0.05); compared with the I/R group, each of the OSAHS hypoxia groups showed an increased number of beclin1 immune-positive cells at each time point (P<0.05); compared with the IH+I/R group, the inhibitor group also showed an increased number of beclin1 immune-positive cells at each time point (P<0.05). (See Table 2 and Figure 2)

### RT-PCR

#### mTOR

Compared with the SO group, the I/R group showed remarkably enhanced expression of mTOR protein at each time point (6h and 24h) (P<0.05); compared with the I/R group, the IH+I/R group showed enhanced expression of mTOR protein at each time point (6h and 24h) (P<0.05); compared with the IH+I/R group, the inhibitor group showed down-regulated expression of mTOR protein at each time point (6h and 24h) (P<0.05). (See Table 3 )

**Table 3:** Changes of the relative expression levels of mTOR and beclin1 mRNA in the hippocampus of each group (x¯ ± *s*)

Group	N	mTOR		beclin1	
		6h	24h	6h	24h
SO group	5	1.00±0.00	1.00±0.00	1.00±0.00	1.00±0.00
I/R group	5	1.22±0.10*	1.27±0.08*	1.57±0.11*	1.79±0.09*
IH+I/R group	5	1.65±0.06*^△^	1.73±0.07*^△^	2.37±0.14*^△^	2.54±0.24*^△^
Inhibitor group	5	1.32±0.07*^△Δ^	1.43±0.06*^△Δ^	2.87±0.10*^△Δ^	2.94±0.15*^△Δ^

Note: *P<0.05, compared with the SO group; ^△^*P*<0.05, compared with the I/R group; ^Δ^*P*<0.05, compared with the IH+I/R group

#### Beclin1

Compared with the SO group, the I/R group showed remarkably enhanced expression of beclin1 protein at each time point (6h and 24h) (P<0.05); compared with the I/R group, each of the OSAHS hypoxia groups showed enhanced expression of beclin1 protein at each time point (6h and 24h) (P<0.05); compared with the IH+I/R group, the inhibitor group also showed enhanced expression of beclin1 protein at each time point (6h and 24h) (P<0.05). (See Table 3)

## DISCUSSION

In the present study, we found intermittent hypoxia preconditioning can aggravate the nerve injury of the global cerebral ischemia-reperfusion model, and the mechanism is associated with the activation of mTOR/autophagy pathway. Our results provided experimental basis for the prevention and cure of OSAHS complicated with ischemic cerebrovascular diseases.

Autophagy widely exists in eukaryotic organisms, which is a phenomenon that cells recognize, degrade and reverse dysfunctional proteins and organelles via the lysosome pathway, stimulated by physiological or pathological factors. It is the third manner of death besides necrosis and apoptosis [8–9]. The results of the study showed that compared with the I/R group, the IH+I/R group showed aggravated structural damage of neurons and a decreased survival density of neurons at each time point (6h and 24h) as well as enhanced expression of beclin1 protein detected by immunohistochemical staining and RT-PCR, which indicated that autophagy exists in the process of pure cerebral ischemia-reperfusion, and intermittent hypoxia can further activate autophagy and worsen neuron loss after cerebral ischemia. These findings are consistent with existed research results at home and abroad. Liu [10] built the intermittent hypoxia mouse model and observed the expression of autophagy-associated proteins of the nerve cells in the mouse hippocampus CA1 region after intermittent hypoxia, and the results showed an increased level of LC3II/LC3≡ protein and exacerbated ultrastructural damage of neurons, which indicated that intermittent hypoxia could induce autophagy in nerve cells of the rat hippocampus. Koike et al. [11] found in the ischemia hypoxia mouse model a remarkable rise of the level of LC3-II protein, the formation of autophagosomes in the pyramidal cell layer of the hippocampus and extensive death of hippocampal neurons, which suggested that the activation of autophagy led to enhanced expression of LC3-II and aggravated neuron injury.

mTOR signal pathway plays an important role in central nervous system diseases. For instance, Su [12] treated the mice suffering cerebral ischemia with rapamycin, and found that rapamycin could up-regulate the phosphorylation level of mTOR protein and reduce the cerebral infarct area, which indicated that the protective effect of a trace amount of rapamycin on acute cerebral ischemic injury was correlated with the activation of mTOR signal pathway. Xu [13] established the SAH model with SD rats which were treated with rapamycin, and detected the expression of phosphorylated mTOR by Western blot, and the results showed enhanced activity of mTOR after SAH, which indicated that a trace amount of rapamycin could down-regulate the phorylation level of mTOR protein and reduce the degree of edema and necrosis of brain tissue. Based on our results in this study, immunohistochemical staining and RT-PCR both showed enhanced expression of mTOR protein in the IH+I/R group at each time point (6h and 24h) compared with the I/R group, which indicated that intermittent hypoxia could activate mTOR signal pathway and deteriorate nerve injury after cerebral ischemia. Studies [14–16] showed that stress responses such as hypoxia, ischemia, low-energy, could regulate the activity of mTORC1 via up-regulating AMPK activity, changing the aggregative state of TSC1/2, phosphorylating TSC2 or directly phosphorylating raptor; and inflammatory factors such as TNF-α could trigger similar disaggregation of TSC1/2 through IKKβ or Wnt signal pathway so as to activate mTORC1. Therefore, intermittent hypoxia can activate mTOR signal pathway to worsen cerebral injury by aggravating anoxia, ischemia and the release of inflammatory factors such as TNF-α in the central nervous system after cerebral ischemia. Recent researches show that mTOR kinase is a pivotal regulatory site of autophagy, which closely correlates with the onset and development of autophagy, and inihibiting mTOR activity can up-regulate autophagy [17–18]. Tizon et al. treated the mice suffering cerebral ischemia with cysteine protease inhibitor C and the results showed decreased activity of mTOR signal pathway, up-regulated autophagy and a lower degree of neuronic injury, which suggested that inhibiting mTOR pathway could induce autophagy so as to exert protective effects on cortex neurons of mice [19]. Zhu et al. [20] found that down-regulating phosphorylation of mTOR via blocking P13K pathway with type IP13K kinase inhibitor could inhibit autophagy induced by high glucose and promote apoptosis induced by high glucose, which indicated that mTOR signal pathway plays an important part in autophagy induced by high glucose. Based on our results in this study, immunohistochemical staining and RT-PCR both showed down-regulated expression of mTOR protein and up-regulated expression of beclin1 protein in the inhibitor group at each time point (6h and 24h) compared with the IH+I/R group, which indicated that mTOR inhibitor could suppress the expression of mTOR protein and then promote the occurrence of autophagy.

Several limitations should be mentioned. Firstly, we performed experiment only in *vivo* but not in *vitro*. Secondly, the results may be more stable if we verified the results in cell model. Finally, the interaction between these two proteins was not be analyzed.

In conclusion, intermittent hypoxia can aggravate the nerve injury of global cerebral ischemia-reperfusion via activating mTOR/autophagy pathway.

## MATERIALS AND METHODS

### Materials

Eighty male Wistar rats were purchased from Beijing Charles River Co., Ltd (license: SCXK).

### Grouping and model preparing

The rats were divided into four groups by the random number method: SO group (n=20), I/R group (n=20), IH7+I/R group (n=20), IH21+I/R group (n=20). Each group was subdivided into two time-point groups: 6h group (n=10) and 24h group (n=10).

In the SO group, vessels were exposed and isolated, electrocoagulation was not performed to the vertebral artery and the common carotid artery was not clipped. In the I/R, IH7+I/R and IH21+I/R group, rat models of global cerebral ischemia were performed with improved Pulsinelli four-vessel occlusion method, and the procedures were as follows: the rats received anesthesia with 10% chloral hydrate and then were fixed at prone position; the back of the occipitalia was cut open in the middle to expose the double flank holes; the preheated electrocoagulation needle was inserted (each time lasted for 2-4s) to seal the bilateral vertebral arteries; the rats were then placed on the surgery board in lying position, and the neck was cut open in the middle to isolate the bilateral common carotid arteries; twenty-four hours later, the bilateral common carotid arteries were simultaneously clipped for 15min to cause global cerebral ischemia.

From 8:00am-15:00pm every day, the rats in the I/R, IH7+I/R and IH21+I/R group stayed in the hypoxic chamber where nitrogen and air alternated with one circle lasting for 120s. Nitrogen entry continued for 30s to maintain an oxygen concentration of 5-21% internally. A digital oxygen meter was employed to monitor the internal oxygen concentration. The whole process continued for 21d.

### HE staining

Five rats were randomly selected from each group 6h and 24h after cerebral ischemia-reperfusion respectively, and fixed by cardiac perfusion with 4% paraformaldehyde. Then brain was harvested after decollation to cut out brain tissue from optic chiasma to cerebral transverse fissure. After the procedures of dehydration, paraffin embedding, sectioning, deparaffinating, xylene clearing and HE staining were performed routinely, the sections were observed under the optical microscope (40×10).

### Immunohistochemical staining

The above-mentioned brain tissue sections were routinely deparaffinated, blocked with 0.3% H_2_O_2_ for 10min and subjected to heat induced epitope retrieval for 1.5 min. After anti-beclin1 and anti-mTOR antibody was added, the sections were incubated in the wet box at 37°C for 30min. Then secondary antibody was added, followed by incubation in the 37°C incubator for another 40min. Next, after DAB development, dehydration, clearing and sealing with neutral balsam, the sections were observed under the microscope. Quantitative analysis of positive rates: five sections were selected for analyzing each index, and five view fields were randomly selected in the hippocampus on each section under the optic microscope (400X), and positive cells of each view field were counted with the computer.

### RT-PCR

Five rats were randomly selected from each group 6h and 24h after cerebral ischemia-reperfusion respectively. Tissue (50~100mg) was collected from the hippocampus CA1 region of the rats and then well grinded in 1mL Trizol reagent to extract total RNA. OD260/280 values were measured with Rotor-Gene3000 fluorogenic quantitative PCR amplifier, and RNA concentrations were calculated with OD260 values. RNA was then stored at -80°C. Detection procedures: the forward and reverse primers of mTOR were GGTGGACGAGCTCTTTGTCA and AGGAGCCCTAACACTCGGAT; the forward and reverse primers of beclin1 were CTCTCGT CAAGGCGTCACTTC and CCTTAGACCCCTCCATT CCTCA; RT-PCR reactions: Stage 1, 2 (reverse transcription reactions): Reps: 1, 42°C 50min, 95°C10sec; Stage 3 (PCR reactions): Reps: 45, 95°C 15s, 56°C 20s; Stage4 (dissociation curve analysis): Dissociation Protocol.

### Statistical analysis

A database of the data obtained in the study was established using Excel 2003 software. All data were shown with x¯ ±s. One-way analysis of variance for repeated data was performed with SPSS17.0 software. P<0.05 indicated significant difference.

## References

[1] Deng XZ, Liu B, Li Y (2014). Research Progress of Obstructive Sleep Apnea Hyponea Syndrome and Hypertension. Advances in Cardiovascular Diseases.

[2] Cho ER, Kim H, Seo HS, Suh S, Lee SK, Shin C (2013). Obstructive sleep apnea as a risk factor for silent cerebral infarction. J Sleep Res.

[3] Wang HY, Xu E (2012). Dual roles of autophagy in cerebral ischemia. International Journal of Cerebrovascular Diseases.

[4] Liu L, Fang YQ, Xue ZF, He YP, Fang RM, Li L (2012). Beta-asarone attenuates ischemia-reperfusion-induced autophagy in rat brains via modulating JNK, p-JNK, Bcl-2 and Beclin 1. Eur J Pharmacol.

[5] Sengupta S, Peterson TR, Sabatini DM (2010). Regulation of the mTOR complex 1 pathway by nutrients, growth factors, and stress. Mol Cell.

[6] Lin Z, McDermott A, Shao L, Kannan A, Morgan M, Stack BC, Moreno M, Davis DA, Cornelius LA, Gao L (2014). Chronic mTOR activation promotes cell survival in Merkel cell carcinoma. Cancer Lett.

[7] Cao ZB (2012). MTOR signaling pathway in the role and mechanism of brain ischemia. Zheng Zou Da Xue.

[8] Ding H, Tang YH, Huang XP (2015). The role of autophagy in cerebral ischemic injury. Chin Pharm Bull.

[9] Ropolo A, Bagnes CI, Molejon MI, A Lo Re, Boggio V, Gonzalez CD, Vaccaro MI (2012). Chemotherapy and autophagy-mediated cell death in pancreatic cancer cells. Pancreatology.

[10] Liu HY, Chen R, Li YN, Zhang YL, Liu CF Chronic intermittent hypoxia area in mice hippocampal CAl neurons autophagy. Chin J Tuberc Respir Dis.2011;.

[11] Koike M, Shibata M, Tadakoshi M, Gotoh K, Komatsu M, Waguri S, Kawahara N, Kuida K, Nagata S, Kominami E, Tanaka K, Uchiyama Y (2008). Inhibition of autophagy prevents hippocampal pyramidal neuron death after hypoxic-ischemic injury. Am J Pathol.

[12] Su FY (2011). Trace rapamycin to study the protective effect and mechanism of ischemic brain damage. Second Military Medical University.

[13] Xu X (2014). MTOR signaling pathway in the role of early brain injury after subarachnoid hemorrhage and mechanism research. Su Zhou Da Xue.

[14] He JY, Liu F (2014). MTOR signaling pathway major diseases associated with aging and aging. Progress in Biochemistry and Biophysics.

[15] Lee DF, Kuo HP, Chen CT, Hsu JM, Chou CK, Wei Y, Sun HL, Li LY, Ping B, Huang WC, He X, Hung JY, Lai CC, Ding Q, Su JL, Yang JY, Sahin AA, Hortobagyi GN, Tsai FJ, Tsai CH, Hung MC (2007). IKK beta suppression of TSC1 links inflammation and tumor angiogenesis via the mTOR pathway. Cell.

[16] Thomas GV, Tran C, Mellinghoff IK, Welsbie DS, Chan E, Fueger B, Czernin J, Sawyers CL (2006). Hypoxiainducible factor determines sensitivity to inhibitors of mTOR in kidney cancer. Nat Med.

[17] Singh BN, Kumar D, Shankar S, Srivastava RK (2012). Rottlerin induces autophagy which leads to apoptotic cell death through inhibition of PI3K /Akt /mTOR pathway in human pancreatic cancer stem cells. Biochem Pharmacol.

[18] Zhao MM, Zhao YB, Luo P, Li SZ, Xu HX, Huo K, Zhang L, Liu WB, Wu Y, Jiang XF, Yue F, Liu ZB, Fei Z (2012). PI3K/Akt signal pathway of autophagy induced by mechanical damage of neurons. The neurosurgical disease research magazine.

[19] Tizon B, Sahoo S, Yu H, Gauthier S, Kumar AR, Mohan P, Figliola M, Pawlik M, Grubb A, Uchiyama Y, Bandyopadhyay U, Cuervo AM, Nixon RA (2010). Induction of autophagy by cystatin C: a mechanism that protects murine primary cortical neurons add neuronal cell lines. PLOS One.

[20] Zhu JL, Ma TA, Chen XH, Yang Q, Ding GH (2013). High glucose increases podocyte autophagy through PI3K-AKT-mTOR signaling pathway. Chinese Journal of Nephrology.

